# Respiratory Hospitalizations and Their Relationship with Air Pollution Sources in the Period of FIFA World Cup and Olympic Games in Rio de Janeiro, Brazil

**DOI:** 10.3390/ijerph18094716

**Published:** 2021-04-28

**Authors:** Élida Campos, Carlos Alexandre R. Pereira, Carmen Freire, Ilce F. da Silva

**Affiliations:** 1 Department of Quantitative Methods in Health, National School of Public Health, Oswaldo Cruz Foundation (FIOCRUZ), Leopoldo Bulhões Street, 1480, Rio de Janeiro 21041-210, Brazil; cfreire@ugr.es (C.F.); ilce23@hotmail.com (I.F.d.S.); 2 Interdisciplinary Center for Social Development (Nides), Federal University of Rio de Janeiro (UFRJ), Athos da Silveira Ramos Avenue, University City, Rio de Janeiro 21941-909, Brazil; carlos.rpereira@hotmail.com; 3 Instituto de Investigación Biosanitaria de Granada (ibs.GRANADA), CIBER de Epidemiología y Salud Pública (CIBERESP), Madrid Avenue, 15, 18012 Granada, Spain

**Keywords:** respiratory disease, air pollution, spatial analysis, Bayesian analysis, sports event

## Abstract

Background: From 2010 onwards, the city of Rio de Janeiro has undergone changes related to the 2014 FIFA World Cup and the 2016 Olympic Games, potentially affecting the respiratory health of inhabitants. Thus, the spatial distribution of respiratory hospitalizations (2008–2017) and the relationship between this outcome and potential air pollution sources in the city of Rio de Janeiro (2013–2017) were evaluated. Methods: An ecological study was performed using the Bayesian model with multivariate Poisson regression for the period of the sporting events (2013–2017). The outcome was the ratio of hospitalizations for respiratory diseases by the population at risk. Data analysis was performed in the total population and by sex and age group. The air pollution-related variables included industrial districts, traffic density, tunnel portals, a seaport, airports, and construction/road work. Results: All explanatory variables, except tunnel portals, were associated with an increase in the outcome. Construction/road work showed a greater magnitude of association than the other pollution-related variables. Airports were associated with an increased hospitalization ratio among the ≥60 year-old group (mean = 2.46, 95% credible intervals = 1.35–4.46). Conclusion: This study allows for a better understanding of the geographical distribution of respiratory problems in the city of Rio de Janeiro. Present results may contribute to improved healthcare planning and raise hypotheses concerning exposure to air pollution and respiratory hospitalizations.

## 1. Introduction

Air pollution is the result of both human activities and natural emissions, and its main constituents are particulate matter (PM), ozone (O_3_), carbon monoxide (CO), nitrogen oxide (NO_x_), sulfur dioxide (SO_2_), and acid aerosols [1]. Heavy industries, vehicles, urbanization, construction, and demolition works act as major human sources of primary air pollutants. Natural emission sources include sea spray, spontaneous combustion of biomass, and volcanoes, among others [2]. In Brazil, vehicular traffic is the main anthropogenic source of air pollution, accounting for 32% of total PM emissions, followed by industrial activities contributing to 19% of PM emissions [3]. Among factors influencing vehicular traffic pollution dispersion in urban areas, road tunnels are particularly important since they hamper the dispersion of traffic pollutants, and tunnel portals act as sources of emission of the concentrated air pollutants [4,5]. Construction and demolition are also important sources of PM in urban areas and may have a relevant impact on air quality, with potential health effects among workers and subjects residing nearby [6,7].

PM is classified according to its size as inhaled coarse particles (aerodynamic diameter between 2.5 and 10 μm), fine respirable particles (<2.5 μm), and ultrafine particles (<0.1 μm) [8]. Depending on their size, particles may deposit in the nasopharyngeal or tracheobronchial region, enter the respiratory tract, deposit in bronchioles and lung alveoli, penetrate the lung tissue, and reach the bloodstream [2]. PM is composed of several potentially toxic elements such as metallic elements (e.g., lead, nickel, manganese, chromium, cadmium), which are present primarily in the coarse fraction of particles [8]. Human exposure to air pollution (especially fine particles) has been associated with increased risk of acute and chronic health effects, such as cardiovascular and respiratory diseases [9], being a significant public health risk factor. Respiratory adverse outcomes associated with air pollution include chronic obstructive lung disease, pneumonia, lung cancer, cystic fibrosis, idiopathic lung fibrosis, allergy, bronchitis, rhinitis, and asthma [8,9,10,11,12,13,14]. In this line, several Brazilian and international studies have shown that increased levels of air pollution from industrial activities, mining, road traffic, burning, and construction, among other sources, ae related to increases in rates of hospital attendance [15,16,17,18], hospitalization [13,19,20,21,22], and mortality [13,23] from respiratory diseases. Some of these studies used spatial data aggregation [21,22], which allows for the estimation of ecological associations between potential air pollution sources and the geographical distribution of disease-related outcomes. 

According to data collected from air quality monitoring stations, the city of Rio de Janeiro, in Southeast Brazil, has an air quality index frequently classified as good or acceptable [24]. However, PM (2.5–10 μm) and especially O_3_ air levels in the city have been frequently found to be above the limits set by national (140 mg/m^3^) and international standards (100 mg/m^3^) [25,26]. The city of Rio de Janeiro has a fleet of 3,085,311 vehicles [27], five industrial districts [28,29], and three airports in operation [30]. The city also has a seaport with an operational area of 1 million m^2^ and cargo handling of around 6,102,900 tons [31]. From 2010 onward, Brazil’s largest cities have undergone extensive alterations, including construction and reform of roads, tunnels, stadiums, and other facilities in order to facilitate attendance to two of the most important sporting events hosted in the country, the 2014 FIFA World Cup and 2016 Olympic Games [32]. In the city of Rio de Janeiro, construction projects directly related to the Olympic Games were mainly carried out downtown and in *Barra da Tijuca*, in the western area of the city. The socioeconomic impacts of such events have been previously studied [32], but data on the potential impact of these interventions on population health in the city are lacking. With this background, the aims of the present study were as follows: (1) to examine the spatial distribution of the ratio of hospitalization for respiratory disease in the city of Rio de Janeiro in the 10-year period between 2008 and 2017; and (2) to explore the relationship between respiratory hospitalizations and potential sources of air pollution, including construction/road work, carried out in the city because of sporting events during the period from 2013 to 2017.

## 2. Materials and Methods

### 2.1. Study Design

First, a descriptive analysis of hospitalization for respiratory disease using data from 2008 to 2017 was performed. Sequence charts were depicted to examine how the hospital admissions by respiratory causes had occurred over time. The ratio of hospitalization for respiratory disease was calculated by dividing the number of hospitalizations in the period from January 2008 to December 2017 by the total population in same period. However, the data from 2008 to 2013 presented were low quality with missing values. A peak could be observed in 2009–2010, likely related to the epidemic by the H1N1 influenza A virus subtype (H1N1). The H1N1 epidemic lasted from March 2009 to August 2010, when the World Health Organization (WHO) declared the post-pandemic phase’s start [33]. During this period, Brazil registered a high number of severe acute respiratory syndrome (SARS) cases, with H1N1 being the cause of 70% of SARS cases in 2009 [33]. 

**Figure 1:**
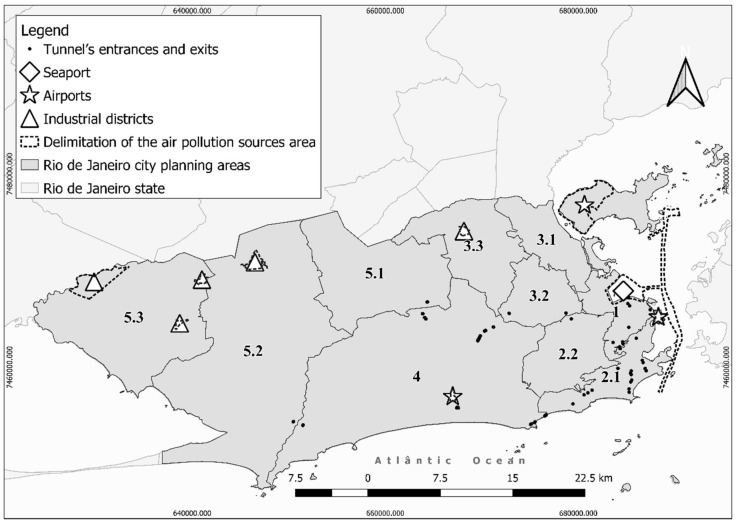
Spatial location of potential air pollution sources and delimitation of planning areas, the city of Rio de Janeiro, Brazil.

Second, an ecological study was carried out to explore the relationship between the ratio of hospitalization for respiratory disease in the city of Rio de Janeiro and various potential explanatory variables, including area covered by industry, vehicular traffic density, number of tunnel portals, construction/road work, and the presence of a seaport and airports, using the city planning areas (PAs) as the geographic unit of analysis (Figure 1). To avoid distortions due to the peak of cases observed in 2009–2010, and considering the relevance of the last five years of the chosen period (i.e., 2013–2017) regarding the intensity of construction work in the city, this study focused on hospital admissions for respiratory disease (International Classification of Diseases 10 version [ICD-10], Chapter X: J00-J99) occurring in the period from 2013 to 2017. In the city of Rio de Janeiro, PAs are organized according to administrative regions, which are encompassed by neighborhoods. Thus, the PA1 is composed of the central regions of the city, i.e., the Downtown, *Portuária*, *Rio Comprido*, *São Cristóvão*, *Paquetá,* and *Santa Teresa* administrative regions; the PA2.1 includes the southern administrative regions of *Botafogo*, *Copacabana*, *Lagoa,* and *Rocinha*; and the PA2.2 includes the northern administrative regions of *Tijuca* and *Vila Isabel*. The PAs 3.1, 3.2, and 3.3 are distributed throughout the northern administrative regions (PA3.1: *Ramos*, *Maré*, *Penha*, *Vigário Geral*, *Ilha do Governador,* and *Complexo do Alemão*; PA3.2: *Méier*, *Jacarezinho,* and *Inhaúma*; PA3.3: *Irajá*, *Madureira*, *Anchieta,* and *Pavuna*). PA4 includes the administrative regions of *Jacarepaguá*, *Cidade de Deus,* and *Barra da Tijuca,* while the PAs 5.1, 5.2, and 5.3 include *Bangu* and *Realengo*, *Campo Grande* and *Guaratiba*, and the *Santa Cruz* administrative regions, respectively.

### 2.2. Data Sources and Study Variables

Information on inpatient hospital admissions was obtained for residents, according to PAs. These data are openly accessible at the Health Information System of Rio de Janeiro Municipality Department, through the TabNet tool (http://tabnet.rio.rj.gov.br/, accessed on 18 June 2018). Hospitalization data were obtained for the total population of the city and were also stratified by sex (males and females) and by age group, i.e.: 0–4 years, 15–59 years, and ≥60 years. The 0–4 and ≥60 years age groups are the most vulnerable to the respiratory effects of air pollution, since the most severe cases usually occur in people at the extremes of life (children under 5; adults >60 years). Therefore, these age groups are considered as “sentinel groups” for respiratory diseases that lead to hospitalization [34,35]. Work status also influences the exposure pattern [34]. The group of 15–59 years comprises an employed or potentially active population, which is likely to be more exposed to pollutants from the construction/road work carried out in the city for sporting events, in addition to their exposure at the work place.

In 2011, the number of hospital admissions for respiratory diseases recorded for the PA5.3 was null, most probably because there was a fire in the Pedro II Hospital in October 2010. This hospital is in the central region of the Santa Cruz neighborhood and is the main health center of the area. After the fire, the Pedro II Hospital was reopened in June 2012 [36]. Therefore, hospitalization data in this PA for the years 2010–2012 was corrected by using the arithmetic mean of the previous 2–4 years proportional to the number of months that the hospital was closed (2010: mean between 2008 and 2009 proportional to 2 months; 2011: mean of 2008, 2009, and 2010 proportional to 12 months; 2012: mean of 2008, 2009, 2010, and 2011 proportional to 6 months). 

Information on the population of the city of Rio de Janeiro by sex and age was obtained from the demographic census conducted in 2010 for all administrative regions [37]. For the remaining years of the study period, we used population estimates available at the Brazilian Institute of Geography and Statistics [38] and the Health Information System website [39]. For non-census years, population data were estimated by applying the population proportion of each PA in 2010 to the total population of the city for each year. Age-specific population data were not available for 2016 and 2017, and thus, the moving average of the age-specific population in the previous three years was used. 

Geographical information on administrative delimitation of the city and roads was obtained for the year 2018 in shapefile format from the IBGE website (https://downloads.ibge.gov.br/downloads_geociencias.htm, accessed on 2 August 2018). Spatial layers were then obtained for all PAs, tunnel entrances and exits, airports, and seaport areas in 2018 (Figure 1). Regarding information on tunnels, we calculated the number of entrances and exits in each PA and categorized them into quintiles. The “airport” and “seaport” variables were treated as dichotomous, i.e., the presence or absence in each PA. Traffic density data (24-h average number of vehicles in working days) were obtained from the Engineering and Traffic Company of Rio de Janeiro (CET-Rio) website (http://prefeitura.rio/web/cetrio, accessed on 22 October 2018) for the year 2017, or 2014 when data for 2017 were not available. Bus Rapid Transit (BRT) routes are not monitored by the CET-Rio, and thus, current BRT traffic count data available at the BRT website (http://brtrio.com/, accessed on 14 November 2018) were used. On the basis of the average traffic density in working days, weighted quartiles were computed, generating four groups of roads/streets/avenues by traffic density. A zero weight was attributed to streets lacking information on traffic density. When a street had more than one monitoring point, the arithmetic mean of the different measurements was considered. Next, the weights attributed to roads within each PA were summed and, finally, this sum was stratified into quintiles to have a comparative measure of traffic among the PA as follows: 1–55, 56–68, 69–85, 86–95, and >95. These values were used as weights and are dimensionless.

Regarding construction/road work, the main works done in the city between 2013 and 2017 due to the sporting events of the 2014 World Cup and 2016 Olympic Games were considered, including the *Transolímpica*, *Transcarioca,* and *Transbrasil* roads, the Olympic and Paralympic village, the *Vila Autódromo* community demolition, the reform of the football stadiums *Jornalista Mario Filho* and *Nilton Santos* (better known as “*Maracanã*” and “*Engenhão*”, respectively), construction of the Metropolitan Line Four, construction of the *Joá* double viaduct, the *Perimetral* viaduct demolition, Light Rail Vehicle construction, tunnels construction, and seaport area revitalization works. This variable was dichotomous, i.e., the presence or absence of major construction/road works in the PAs. Industrial districts were delimited using the information available at the website of the Industrial Development Company (CODIN) of Rio de Janeiro state (http://www.codin.rj.gov.br/, accessed on 12 June 2018). For each PA, the percentage of area covered by industrial districts was calculated. 

The QuantumGis program (3.2.3 version) was used to create the maps and manipulate the geographical information. All data used in this study were obtained from public sources, and thus, ethics approval was not required.

### 2.3. Statistical Analyses 

First, the crude ratio of hospitalization for respiratory disease was calculated for the total population and according to sex and age group. Subsequently, sequence charts were plotted to perform a descriptive analysis of the crude ratios of hospitalization for respiratory diseases over the period 2008–2017. The charts were created using the Statistical Package for Social Sciences (SPSS) for Windows program (SPSS Inc. Released 2008. SPSS Statistics for Windows, 17.0 version. Chicago: SPSS Inc). Finally, Bayesian models were created for modeling the relationship between the potential explanatory variables and the ratio of hospitalization for respiratory disease. Regarding the association with air pollution-related variables, a Poisson regression with explanatory variables as fixed effects was performed. Models included a spatial random effect term to adjust for the proportion of the outcome variation not explained by the potential explanatory variables.

Bayesian analysis is based on Bayes’ theorem that describes the probability of an event, based on prior knowledge of conditions that might be related to the event, according to the following equation:

(1) $$P\left(H\vee data\right)=\left(P\left(data\vee H\right)xP\left(H\right)\right)/\left(P\left(data\right)\right)$$

where *P*(*data*|*H*) is the likelihood function and describes the probability of the data under the H hypothesis; *P*(*H*) is the prior probability and describes what is known about the H hypothesis; *P*(*data*) is the so-called normalization constant and serves to place the result on a 0–1 scale; and *P* (*H*|*data*) describes the posterior distribution obtained after collecting all data, that is the probability of the *H* hypothesis conditional on the observed data.

One simple way to obtain the posterior probability is using a simplification of the equation above, so that posterior probability is proportional to the product of likelihood and prior distribution:

*posterior α likelihood* × *prior* (2)

where the likelihood describes the distribution that best fits the observed data and prior distribution describes the distribution of data known a priori. In the adjusted Poisson model regression, the likelihood was expressed as:

*P* (*hospitalization data*|*H*) ~ *Poisson* (*µ*) (3)

where:

μ = log (tot[i]) + β[1] + β[2] * airport[i] + β[3] * industry[i] + β[4] * traffic[i] + β[5] * construction[i] + β[6] * seaport[i] + β[7] * tunnel[i] + s[i](4)

where log(tot[i]) is the logarithmic denominator of the ratio of hospitalization, which in the Poisson regression is considered as a covariate with a regression coefficient fixed in 1 (offset term); β[1] is the constant term, and β[2]–β[7] are the beta coefficients of the explanatory variables, i.e., “airport” (presence/absence), “industry” (% area covered by industrial districts), “traffic” (sum of weights of traffic density categorized into quintiles), “construction” (presence/absence of major construction and/or road work), “seaport” (presence/absence), and “tunnel” (number of tunnel entrances and exits categorized into quintiles). The *s* term describes the spatial random effect. 

In Bayesian analysis defining the prior distributions for all model parameters is required, including the random effect term that describes the spatial component (*s*). A uniform distribution (“flat distribution” in OpenBugs language) was set for β_1_, i.e., for the constant term. In the flat distribution, all “x” terms in the model have a constant value, thus being an improper distribution. For the other β coefficients in the equation, a non-informative normal distribution (0, 0.1) was considered. The spatial component (*s*) was treated as an adjustment variable with a conditional autoregressive model (CAR) prior to distribution. In the CAR model, it is assumed that the spatial dependence can be determined by a conditional distribution for *s,* so that the autoregressive parameters can be determined for a multivariate normal distribution, as follows:

*s*[1:*N*] ~ *car.normal*(*adj*[], *weights*[], *num*[], *tau*) (5)

where *adj*[] is the neighbor list of each PA; *weights*[] describes the weights of the spatial influence between PAs; *num*[] is the total number of neighbors of each PA; and *tau* describes the precision, which is the square of the inverse of the standard deviation.

[*sigma* = sqrt(1/*tau*)]. (6)

In the CAR model, a value equal to 1 was attributed to neighborhood weight, thus maximizing the general spatial correlation. The resulting model is called an “intrinsic conditional autoregressive” (ICAR) model. This approach simplifies the CAR model regarding the assumption that the weights must be symmetric (Wij = Wji), but it requires the definition of a distribution for the *tau* parameter. Since the *gamma* distribution is generally used to describe the precision and standard deviation, a non-informative *gamma* distribution (0.01, 0.01) was defined.

Statistical analyses were performed by using the OpenBUGS program (David Spiegelhalter, Andrew Thomas, Nicky Best and Dave Lunn, 3.2.3 version, rev 1012 of 2014. Cambridge CB2 2SR, UK), which uses the Markov chain Monte Carlo (MCMC) method. By using this method, several iterations were run until convergence was reached. To achieve faster convergence, multiple chains (five) were used. A thin equal to 10 was used for reducing the autocorrelation in the sample. In addition, to reduce the autocorrelation between chains, the option ‘over relax’ in the update tool dialogue box was selected. The convergence decision was made through Brooks–Gelman–Rubin (BGR) diagnostic plots analysis (Figure S1) and visual inspection of the posterior distribution parameters. The Brooks–Gelman–Rubin (BGR) diagnostic can be used when two or more chains are generated. For each variable in the model, the BGR makes a comparison between the variance within each chain and the variance among the chains. The more the posterior distribution approximates to the normal distribution, the better results the BGR obtains. A graph with three lines (red, green, and blue lines) is obtained from the BGR analysis. The red line informs about the results of the modeling and it is expected to converge to 1; the green line informs about the variance among the chains, and the blue line informs about the variance within an individual chain. It can be assumed that the convergence has been reached when the red line converges to 1, while the green and blue lines converge to a reasonable stability. It was considered that convergences were reached after 15,000 iterations for the total population: 22,000 for males, 11,000 for females, and 28,000, 5000, and 14,000 iterations for the 0–4, 15–59, and ≥60 year-old groups, respectively. Final regression models were simultaneously adjusted for all potential explanatory variables described above. Associations between explanatory variables and the ratio of hospitalization for respiratory disease were expressed as the exponential of the mean of the posterior beta (β) and its 95% credible interval (95% CI).

## 3. Results

The descriptive analysis of the ratio of hospitalization for respiratory diseases in the PAs of Rio de Janeiro (Figures S2 and S3) showed some inconsistencies, hampering the use of the entire period (2008–2017) in the Bayesian analysis. A peak was observed in 2009–2010 in almost all PAs in the total population, in both males and females (Figure S2), whereas the ratio of hospitalization seemed to be more stable over the last five years of the study period in all groups (Figures S2 and S3).

**Figure 2:**
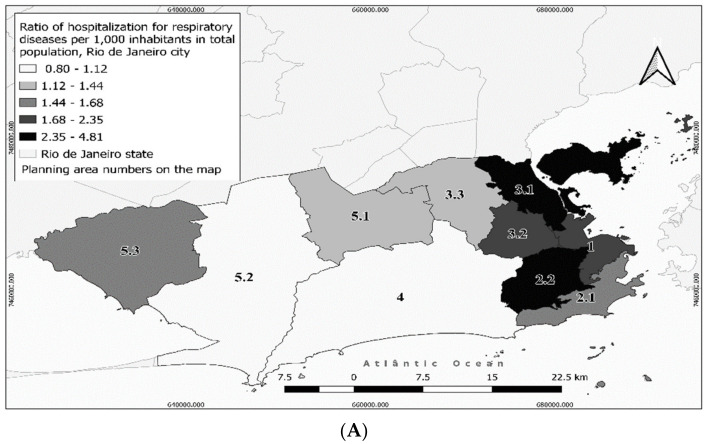

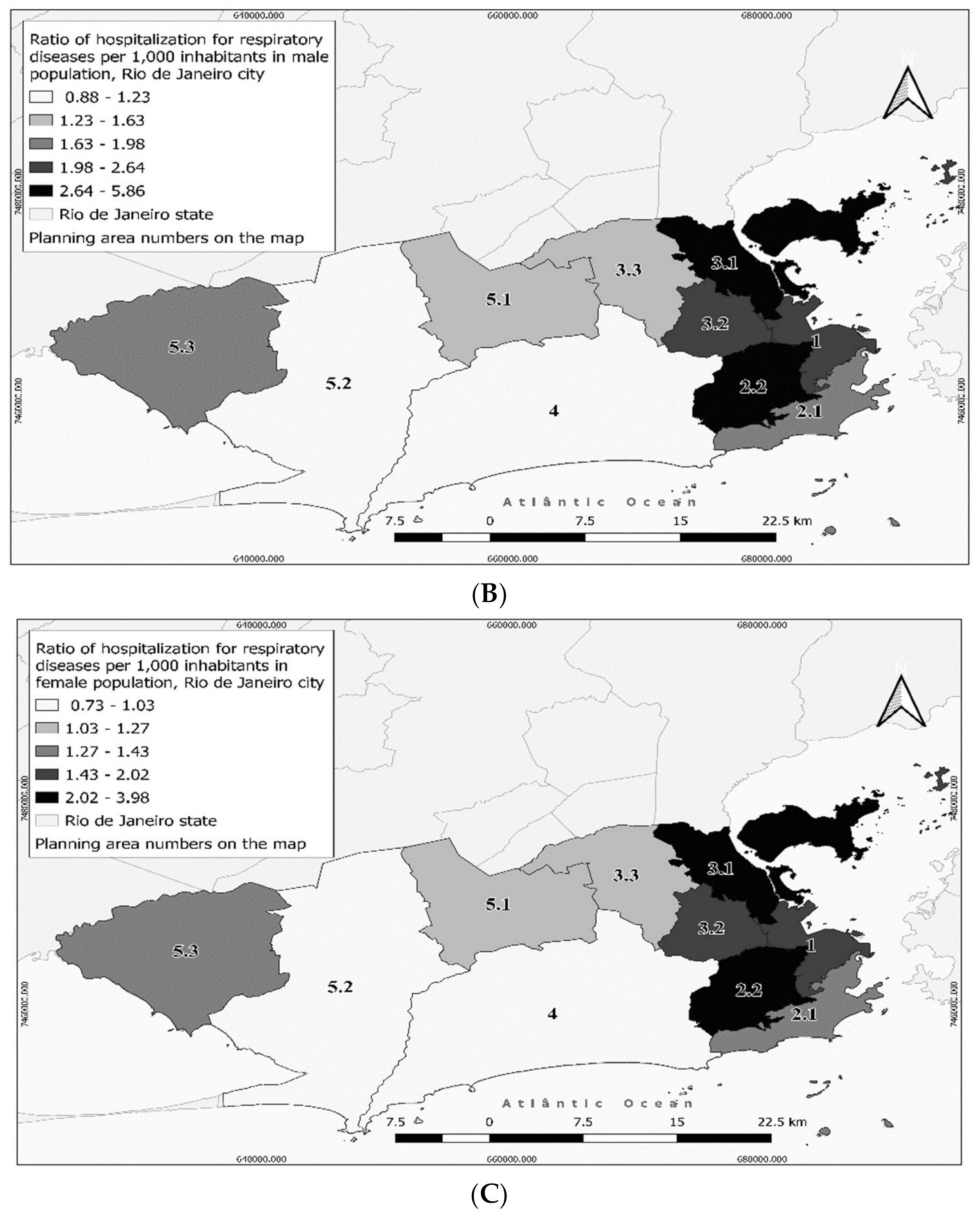
Ratio of respiratory hospitalizations in the city of Rio de Janeiro in 2013–2017: (**A**) general population, (**B**) male population, and (**C**) female population.

**Figure 3:**
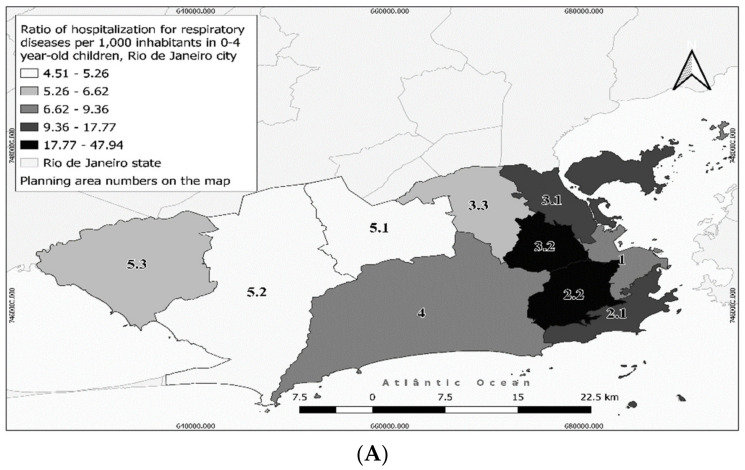

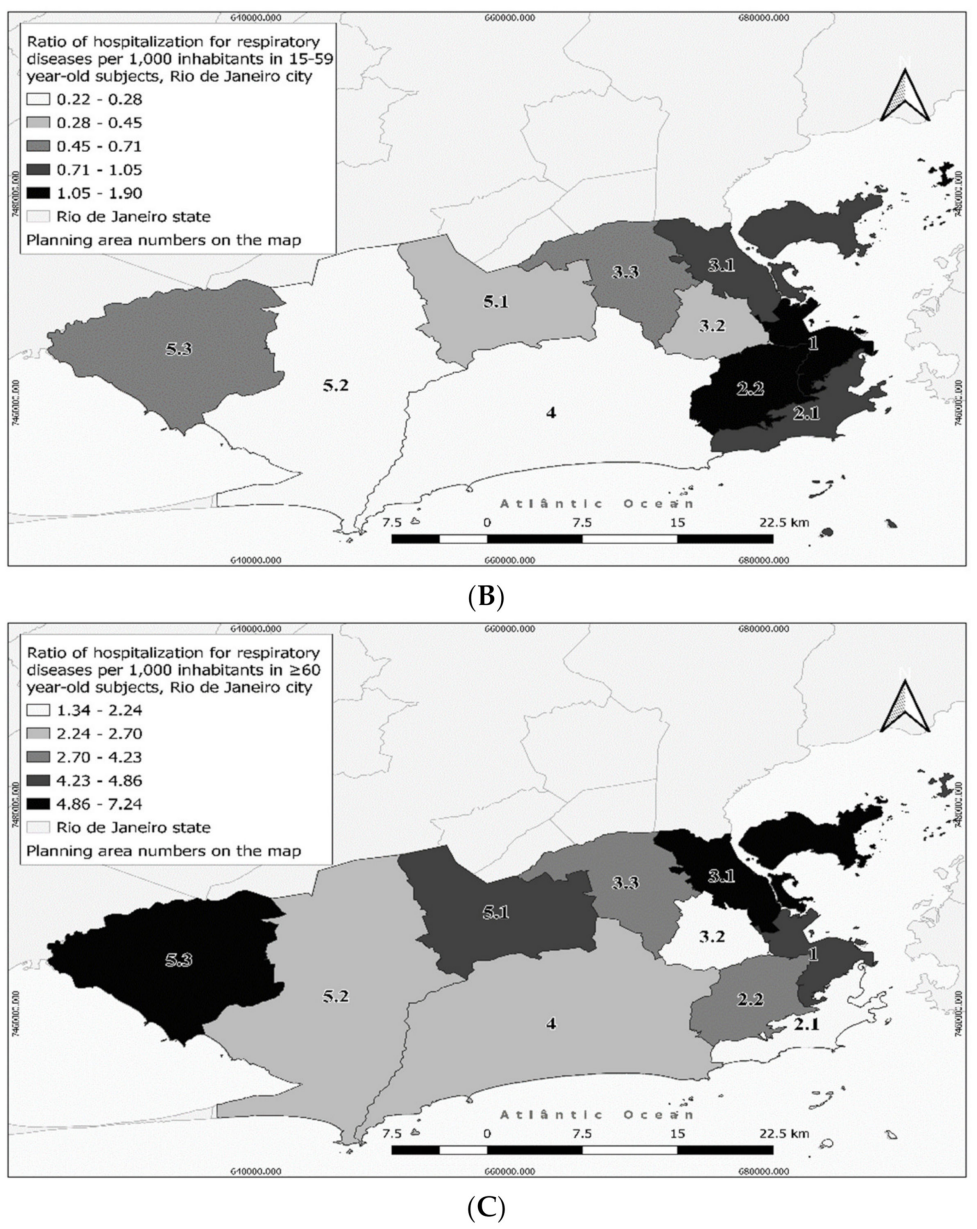
Ratio of respiratory hospitalizations in the city of Rio de Janeiro in 2013–2017: (**A**) 0–4 year-old children, (**B**) 15–59 year-old subjects, and (**C**) ≥60 year-old subjects.

In the period from 2013 to 2017, there were 57,661 hospitalizations for respiratory disease in the city. Figure 2 and Figure 3 show the hospitalization ratio in the PAs. PA2.2 presented the highest ratio of hospitalization for respiratory disease in the total population (4.81 per 1000 inhabitants), followed by PA3.1 (3.15 per 1000 inhabitants) and PA1 (2.11 per 1000 inhabitants) (Figure 2). PA2.2 also had the highest ratio in 0–4-year-old children (47.94 per 1000 inhabitants), 15–59-year-old subjects (1.90 per 1000 inhabitants), males (5.86 per 1000 inhabitants) and females (3.98 per 1000 inhabitants) (Figure 3). In the ≥60-year-old group, the highest hospitalization ratio occurred in PA3.1 (7.24 per 1000 inhabitants) (Figure 3). There are no industrial districts in the PAs with higher hospitalization ratios for respiratory disease, but the PA3.1 has a seaport and an airport. PAs 3.1 and 2.2 had “moderate” traffic density (sum of weights = 56–68 and 86–95, respectively) and up to five tunnel entrances and exits. In both PAs, important construction/road work was carried out during the study period (Table 1), particularly the reform of the *Maracanã* soccer stadium (PA2.2), and the *Transcarioca* and *Transbrasil* road constructions (PA3.1).

Results of multivariate Poisson regression analysis for each explanatory variable are shown in Table 2, which exhibits the mean, the standard deviation, and the 2.5 and 97.5 percentiles for each Beta in the posterior distribution. The presence of airport in the PAs was positively associated with the hospitalization ratio (mean = 2.46; 95% CI = 1.35–4.46) in the ≥60-year-old population. In addition, a high coefficient of association was observed between construction/road works and increased hospitalization ratio in all population groups. The magnitude of the association with construction/road work was higher in men (mean = 3.29; 95% CI = 0.39–16.22), 0–4-year-old subjects (mean = 2.73; 95% CI = 0.11–13.76), and 15–59-year-old (mean = 2.64; 95% CI = 0.37–9.91) subjects. Although the unit is contained in the 95% CI and the amplitude of the CI is very large, associations were mostly positive for all explanatory variables and population groups, except tunnels, based on the mean of the beta coefficient.

## 4. Discussion

In this study, a Bayesian model with multivariate Poisson regression for the period of the sporting events (2013–2017) was fitted. The Bayesian model was chosen as the option for this study because of the small number of PAs and the possibility to perform a model despite the intrinsic classical modeling requirements to drawing multivariate Poisson regression, concerning data variance, for example. When noninformative prior distributions are used, the Bayesian results are very close, generally, to the classical modeling results. Poisson distribution was selected to the likelihood function in line with other studies that presented Poisson models as a most recommended choice to perform linear or additive classical [40] or Bayesian [41] models for relating air pollution and mortality or morbidity. Since the temporal data of explanatory variables was lacking, time-series hierarchical models could not be proceeded. Thus, only analysis with aggregated data could proceed; although this provides information on the magnitude of the relationship between the variables, such an analysis does not inform about the temporal effect on this relationship.

Despite this limitation, this study examined for the first time the relationship between the main sources of air pollution and respiratory hospitalizations in the city of Rio de Janeiro during the period of construction projects directly related to the 2014 FIFA World Cup and 2016 Olympic Games. Construction/road work carried out in PAs of Rio de Janeiro for the international sports events hosted in the city was the potential polluting source most strongly associated with the ratio of hospitalization for respiratory disease, despite the unit being contained in the 95% CI. Besides, airports were related to hospital admissions for respiratory disease in the older population.

Construction and demolition works using concrete release large amounts of coarse PM, but also fine and ultrafine particles [6,42]. Other materials used in construction and road work, such as asphalt pavements, also release PM and gaseous pollutants, including NOx [43]. Respiratory health effects of PM and common urban air pollutants are well-known [8,9,10,11,12,13,14]. The impact of large infrastructure projects on atmospheric pollution is particularly relevant in big cities of developing countries due to the economic relevance of the civil construction sector compared to developed countries [42]. Contrary to expectations for improvements in health due to the Olympic Games’ legacy [44], the results of the present study suggest that construction/road work carried out in the city may have contributed to the increase in respiratory hospitalizations in 2013–2017. However, the potential effect of construction/road work on respiratory hospitalizations could be better understood if information on the intensity or quantity of construction projects was available.

The importance of vehicular traffic management and urban planning in improving air quality was shown, for example, during the Olympic Games in Rio de Janeiro [45]. In this study, there was a positive association between traffic density and the ratio of hospitalization for respiratory disease among the population under 60 years old, but the 95% CI contained the unit. Similar results were observed in a Brazilian ecological study in the city of São Paulo that found a positive association between the risk of hospitalization for respiratory cancer and higher traffic density [21]. Although the ecological design is limited to infer causal relationships, the observed association between traffic density and increased ratio of respiratory hospitalizations in the PAs of Rio de Janeiro corroborates the findings of analytical (individual-level) North American studies showing positive associations between exposure to air pollution from heavy traffic and hospitalization for respiratory diseases [46,47]. For the older population (≥60 years), the observed results suggest that the presence of an airport in the PA may contribute to increased respiratory hospitalizations. The literature on the impact of airports on respiratory health is very limited [48], with one cross-sectional study conducted in the United States showing that subjects living near two of the three investigated airports were at higher risk for respiratory hospitalization compared to subjects living in more distant areas [49].

Some analytical studies investigating populations living near industrial areas reported an increased risk of respiratory disease or mortality concerning exposure to industrial air emissions [20,50,51]. In this sense, the present study may suggest that industrial activities could be a potential risk factor for respiratory symptoms or disease, resulting in a greater risk for hospitalization due to respiratory causes in Rio de Janeiro. The fact that the 95% CI contained the unit could be explained by the inherent limitation of the “industrial district” variable. Information on the type and number of industrial facilities in each industrial district was not available, representing a limitation in this study, because the nature and intensity of air pollution emissions are highly dependent on the type of industrial activity [51]. Similarly, the “seaport” credibility interval contained the unit in the multivariate model. It is known that seaports can contribute to increased air pollution levels [52,53,54]. For instance, an Italian study reported greater asthma-related hospitalizations in children of the industrial seaport area than the expected number of cases in the Lazio region [55]. In the present study, an important part of construction projects was carried out in the seaport area of the city of Rio de Janeiro. To overcome this, an interaction term between construction and seaport was introduced in the model, but the goodness-of-fit model was limited (data not presented). While road tunnels hamper the dispersion of air pollution by acting as a protecting structure [4], they can concentrate air pollutants to 10 times the levels found in open areas, so that individuals who spend large amounts of time inside tunnels (inside the car, bus, or other vehicles) are potentially exposed to higher levels of air pollution [4,5]. Likewise, individuals living near a tunnel entrance or exit could be at higher risk for traffic-related air pollution exposure. Although the 95% CI of the observed association also contained the unit, the present study suggests a possible inverse correlation between tunnels and respiratory hospitalizations. Overall, further studies are needed to explore the potential impact of road tunnels on respiratory health of the population living in areas surrounding tunnels’ entrances and exits.

The hospitalizations for respiratory diseases considered in the present study were restricted to cases attended by the Brazilian Unified Health System (SUS). Although all Brazilian citizens have the right of access to the public healthcare system, it is well known that the SUS is mostly used by individuals of lower socioeconomic status who have limited access to supplementary healthcare services [56]. Thus, data on hospitalizations for respiratory disease in the Health Information System of Rio de Janeiro are more representative of the lower socioeconomic strata of the population. Unfortunately, controlling for socioeconomic status would not have been adequate in our study because of the homogeneous socioeconomic profile of the population attend by SUS.

Ecological studies are subject to the so-called ecological fallacy, which results from inferring that associations at the aggregate level are true at the individual level. Even though ecological studies are useful to raise, for example, the hypothesis that exposure to urban construction works and airports is associated with adverse respiratory outcomes, additional studies focusing on individuals are necessary to test such a hypothesis. Besides the inherent limitations of ecological studies, the lack of information on temporal variation of explanatory variables such as traffic density limited the present study analyses. However, the analysis was restricted to the last five years of the period (2013–2017), thus reducing the misinterpretation derived from the data variation over the period 2008–2012. Additional study limitations include the lack of information on types of industrial activity within industrial districts; lack of meteorological parameters that influence pollutant dispersion, such as wind and temperature, for all the PAs; and the lack of adjustment for individual potential explanatory variables, such as smoking habit, due to the unavailability of smoking prevalence data by PAs. Finally, it is noteworthy that air pollution monitoring data were not available for all PAs, and it was not possible to conduct a dynamic analysis of pollutants’ dispersion. Instead, information on the main air pollution sources was assessed as a potential explanatory variable using a neighborhood effect model.

Despite the study limitations, the present study sheds light on the potential respiratory health effects of construction projects carried out in Rio de Janeiro in 2013–2017. Finally, the present spatial analysis of respiratory hospitalizations as a function of potential air pollution sources could contribute to health planning in the city of Rio de Janeiro.

## 5. Conclusions

Findings of the present study suggest that construction/road work done in the city of Rio de Janeiro to host two major sporting events, as well as the presence of airports, may be related to increased hospitalizations for respiratory diseases. The construction/road work carried out in the city in the period from 2013 to 2017 may have contributed to increased air pollution levels, which could explain the study findings. Spatial analysis of respiratory hospitalizations may allow public health and urban planning managers to better understand the geographical distribution of respiratory problems in the city of Rio de Janeiro and, then, implement pollution control measures. Furthermore, results of this study may contribute to improve healthcare planning according to the demand for health services. This type of analysis could also help to improve public health interventions planning and adequate resource allocation focused on reducing respiratory morbidity and mortality.

It is also expected that this study may contribute to the debate on the quality of available health and environment information in the city of Rio de Janeiro, the possibility of implementation of local monitoring of air quality, as well as the relationship of such information with potential sources of air pollution and health services demands, especially in the most polluted areas. In a developing country with continental dimensions like Brazil, implementing integrated environmental monitoring systems and georeferenced management is a great challenge because of the limited available resources and organizational structure. Moreover, Southern hemisphere countries also face challenges to manage the impacts of the pressure of urbanization and industrialization. The present study brings an opportunity to the Brazilian environmental, health, and scientific communities to explore the local potential of innovation, creation, observation, and analysis to promote sociotechnical arrangements and adaptations that are more compatible with the real scenario.

## Figures and Tables

**Table 1:** Potential air pollution sources in the city of Rio de Janeiro PAs.

PA	Presence of Airport	Industrial District (% Area Coverage)	Traffic Density *	Construction/Road Work	Presence of Seaport	Tunnel’s Entrances and Exits (No.)
1	Yes	0.00	69–85	Yes	Yes	6–15
2.1	Yes	0.00	≥96	Yes	Yes	16–30
2.2	No	0.00	86–95	Yes	No	1–5
3.1	Yes	0.00	56–68	Yes	Yes	0
3.2	No	0.00	69–85	Yes	No	1–5
3.3	No	0.78	86–95	Yes	No	0
4	No	0.00	≥96	Yes	No	>30
5.1	No	0.00	0–55	Yes	No	1–5
5.2	No	2.76	56–68	No	No	1–5
5.3	No	10.2	0–55	No	No	0

PA: Planning area. * Traffic density: sum of weights.

**Table 2:** Association between potential air pollution sources (explanatory variables) and ratio of hospitalization for respiratory disease in the city of Rio de Janeiro PAs (2013–2017).

Explanatory Variables	Mean	SD	2.5P	97.5P	Mean	SD	2.5P	97.5P	Mean	SD	2.5P	97.5P
	**Total population**				**Males**				**Females**			
Airport	1.31	0.62	0.44	2.91	1.30	0.75	0.41	3.05	1.56	1.09	0.49	4.38
Industrial district	1.03	0.17	0.72	1.40	1.05	0.21	0.70	1.54	1.01	0.18	0.66	1.41
Traffic density	1.07	0.19	0.76	1.54	1.09	0.27	0.67	1.68	1.10	0.25	0.73	1.66
Construction/road work	2.76	2.31	0.44	8.67	3.29	4.60	0.39	16.22	1.97	1.49	0.31	5.77
Seaport	1.28	0.90	0.39	3.79	1.25	0.84	0.27	3.47	1.15	0.79	0.22	3.30
Tunnels	0.76	0.18	0.45	1.15	0.75	0.20	0.41	1.20	0.74	0.18	0.38	1.14
	**0–4 years**				**15–59 years**				**≥60 years**			
Airport	1.34	1.26	0.18	4.99	1.33	0.92	0.29	3.63	**2.46**	**1.52**	**1.35**	**4.46**
Industrial district	1.03	0.35	0.52	1.87	1.09	0.25	0.70	1.66	1.05	0.12	0.78	1.29
Traffic density	1.18	0.41	0.62	2.30	1.23	0.31	0.80	2.04	0.96	0.15	0.73	1.35
Construction/road work	2.73	4.13	0.11	13.76	2.64	3.01	0.37	9.91	1.77	1.13	0.03	4.49
Seaport	1.19	1.50	0.14	5.81	2.69	2.37	0.44	8.07	1.01	0.35	0.41	1.78
Tunnels	0.84	0.32	0.38	1.58	0.70	0.20	0.37	1.19	0.73	0.10	0.53	0.95

SD: standard deviation; 2.5P: 2.5% percentile of credibility interval; 97.5P: 97.5% percentile of credibility interval. Bold: credibility interval does not include unit. All models are simultaneously adjusted for all potential explanatory variables.

## Data Availability

Not applicable.

## Supplementary Materials

The following are available online at https://www.mdpi.com/article/10.3390/ijerph18094716/s1, Figure S1: Brooks-Gelman-Rubin (BGR) diagnostic plots—convergence statistic graphs of spatial analysis models with Poisson regression of the estimated association between the pollution sources and the ratio of hospitalization for respiratory diseases in the Rio de Janeiro city planning areas, Brazil (2013–2017), Figure S2: Sequence charts used in descriptive analysis of hospitalization for respiratory diseases in total population and by sex, in the Rio de Janeiro city planning areas (PA), Brazil (2008–2017), Figure S3: Sequence charts used in descriptive analysis of hospitalization for respiratory diseases by age (children: 0–4 years-old; adults: 15–59 years-old, elderly: ≥60 years-old) in the Rio de Janeiro city planning areas (PA), Brazil (2008–2017).
