# Supplementary material for: Respiratory Hospitalizations and Their Relationship with Air Pollution Sources in the Period of FIFA World Cup and Olympic Games in Rio de Janeiro, Brazil
Source: Int J Environ Res Public Health. 2021 Apr 28;18(9):4716. doi: 10.3390/ijerph18094716 (PMC8124488; doi:10.3390/ijerph18094716)

Figure S1: Brooks-Gelman-Rubin (BGR) diagnostic plots - convergence statistic graphs of spatial analysis models with Poisson regression of the estimated association between the pollution sources and the ratio of hospitalization for respiratory diseases in the Rio de Janeiro city planning areas, Brazil (2013-2017).

1.1. Total population

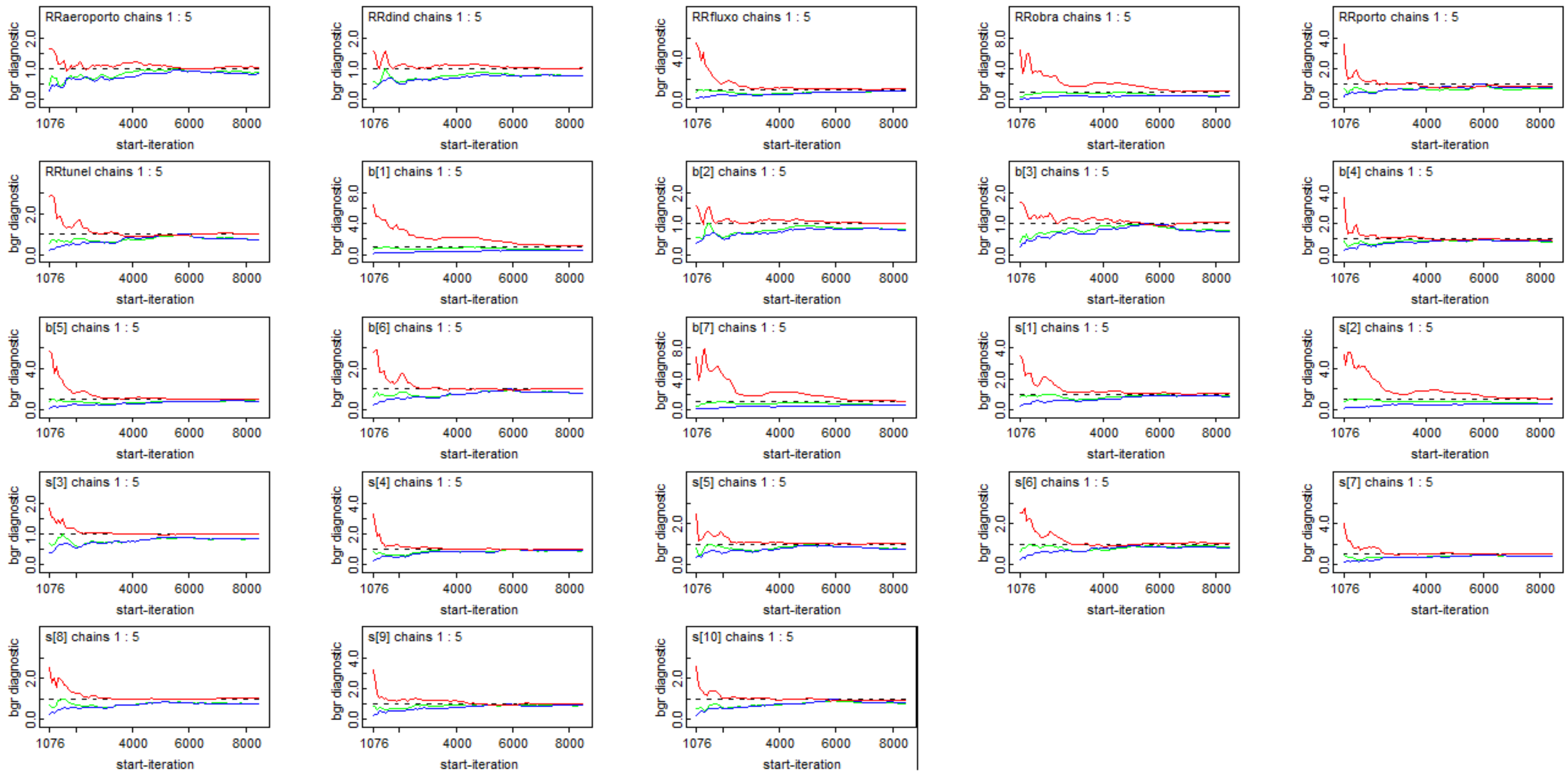

1.2. Men

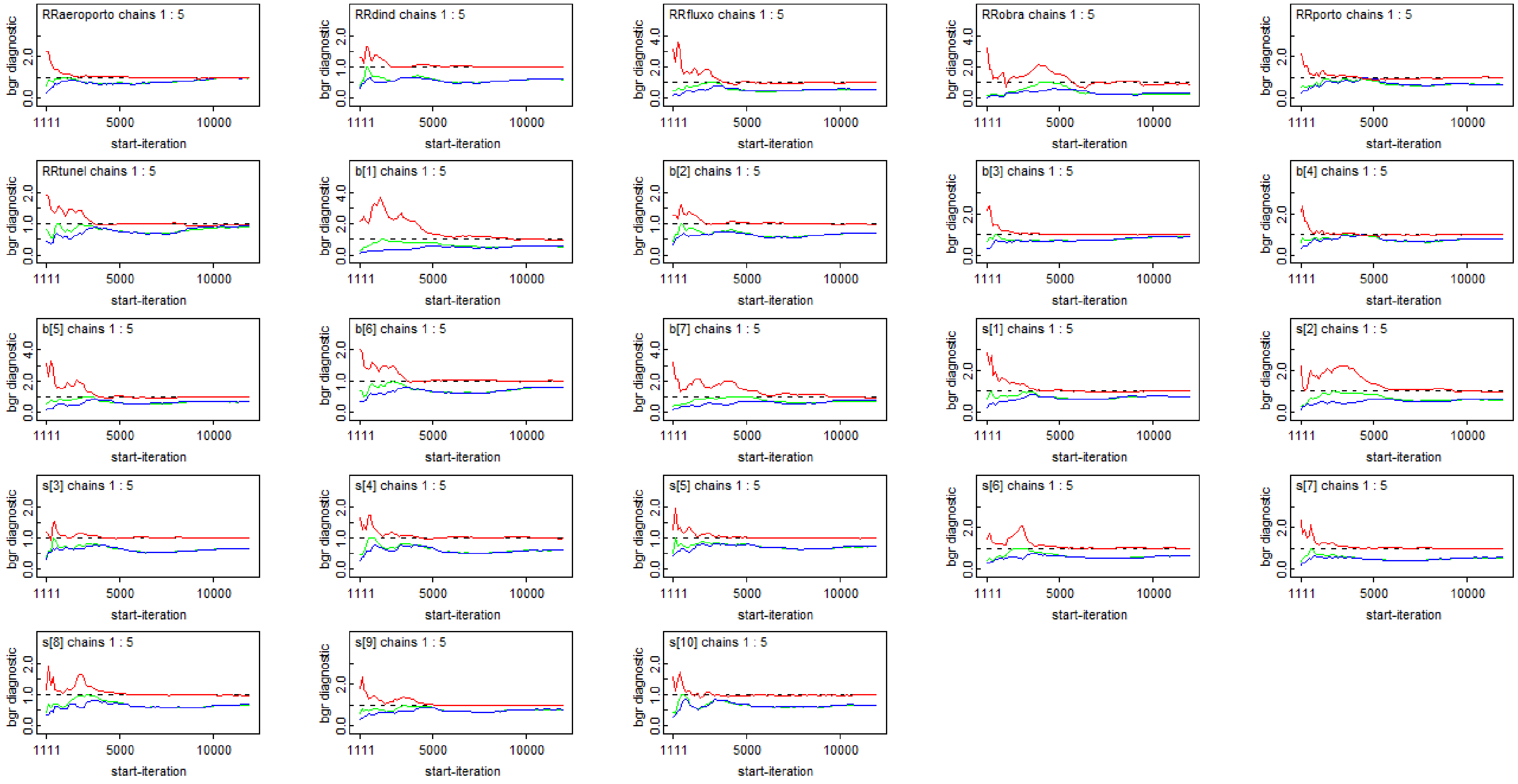

RRaeroporto chains 1 : 5
RRdind chains 1 : 5
RRfluxo chains 1 : 5
RRobra chains 1 : 5
RRporto chains 1 : 5
RRtunel chains 1 : 5
b[1] chains 1 : 5
b[2] chains 1 : 5
b[3] chains 1 : 5
b[4] chains 1 : 5
b[5] chains 1 : 5
b[6] chains 1 : 5
b[7] chains 1 : 5
s[1] chains 1 : 5
s[2] chains 1 : 5
s[3] chains 1 : 5
s[4] chains 1 : 5
s[5] chains 1 : 5
s[6] chains 1 : 5
s[7] chains 1 : 5
s[8] chains 1 : 5
s[9] chains 1 : 5
s[10] chains 1 : 5
bgr diagnostic
start-iteration
1111
5000
10000

1.3. Women

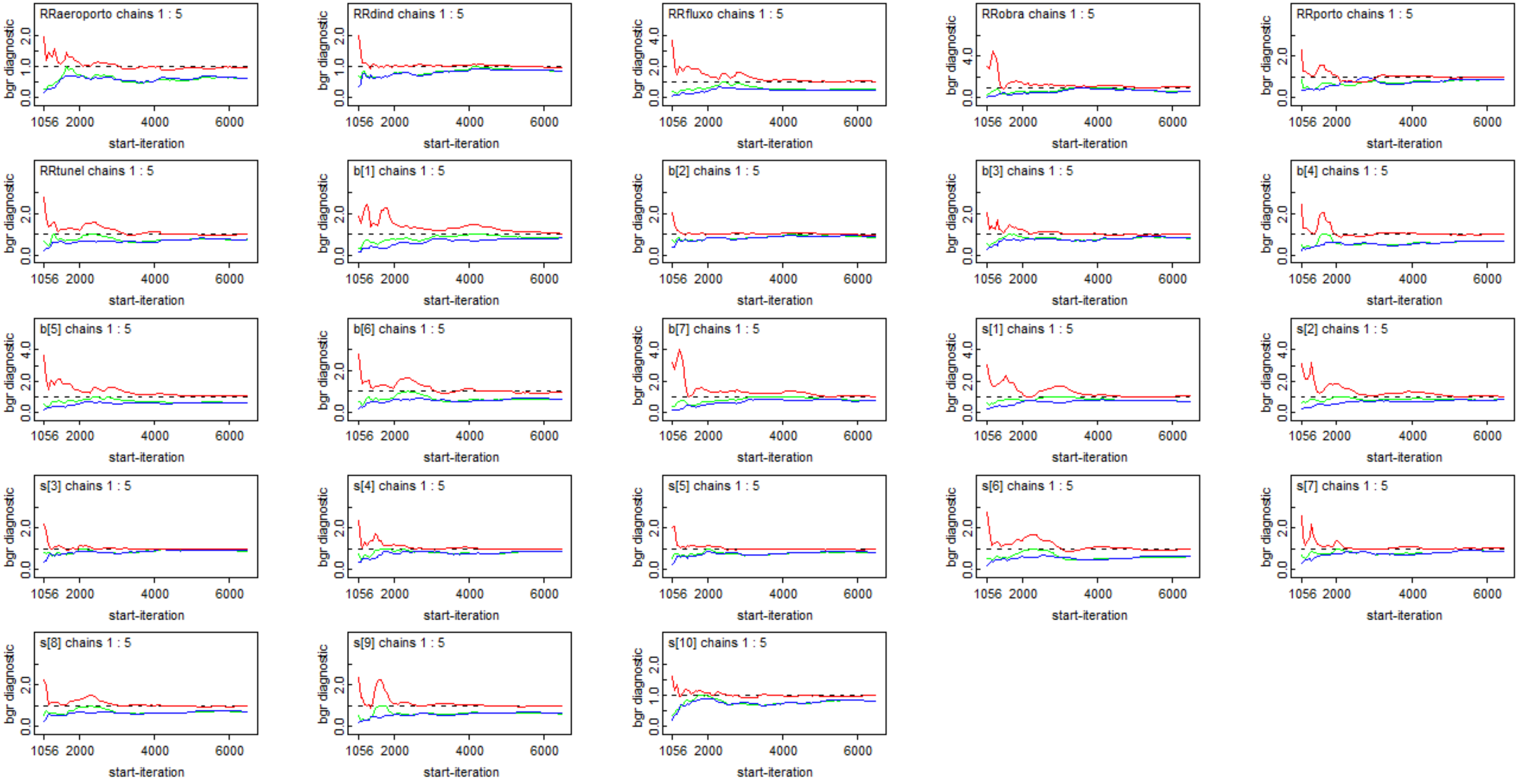

RRaeroporto chains 1 : 5
RRdind chains 1 : 5
RRfluxo chains 1 : 5
RRobra chains 1 : 5
RRporto chains 1 : 5
RRtunel chains 1 : 5
b[1] chains 1 : 5
b[2] chains 1 : 5
b[3] chains 1 : 5
b[4] chains 1 : 5
b[5] chains 1 : 5
b[6] chains 1 : 5
b[7] chains 1 : 5
s[1] chains 1 : 5
s[2] chains 1 : 5
s[3] chains 1 : 5
s[4] chains 1 : 5
s[5] chains 1 : 5
s[6] chains 1 : 5
s[7] chains 1 : 5
s[8] chains 1 : 5
s[9] chains 1 : 5
s[10] chains 1 : 5
bgr diagnostic
start-iteration
1056
2000
4000
6000

1.4. Children (0-4 years-old)

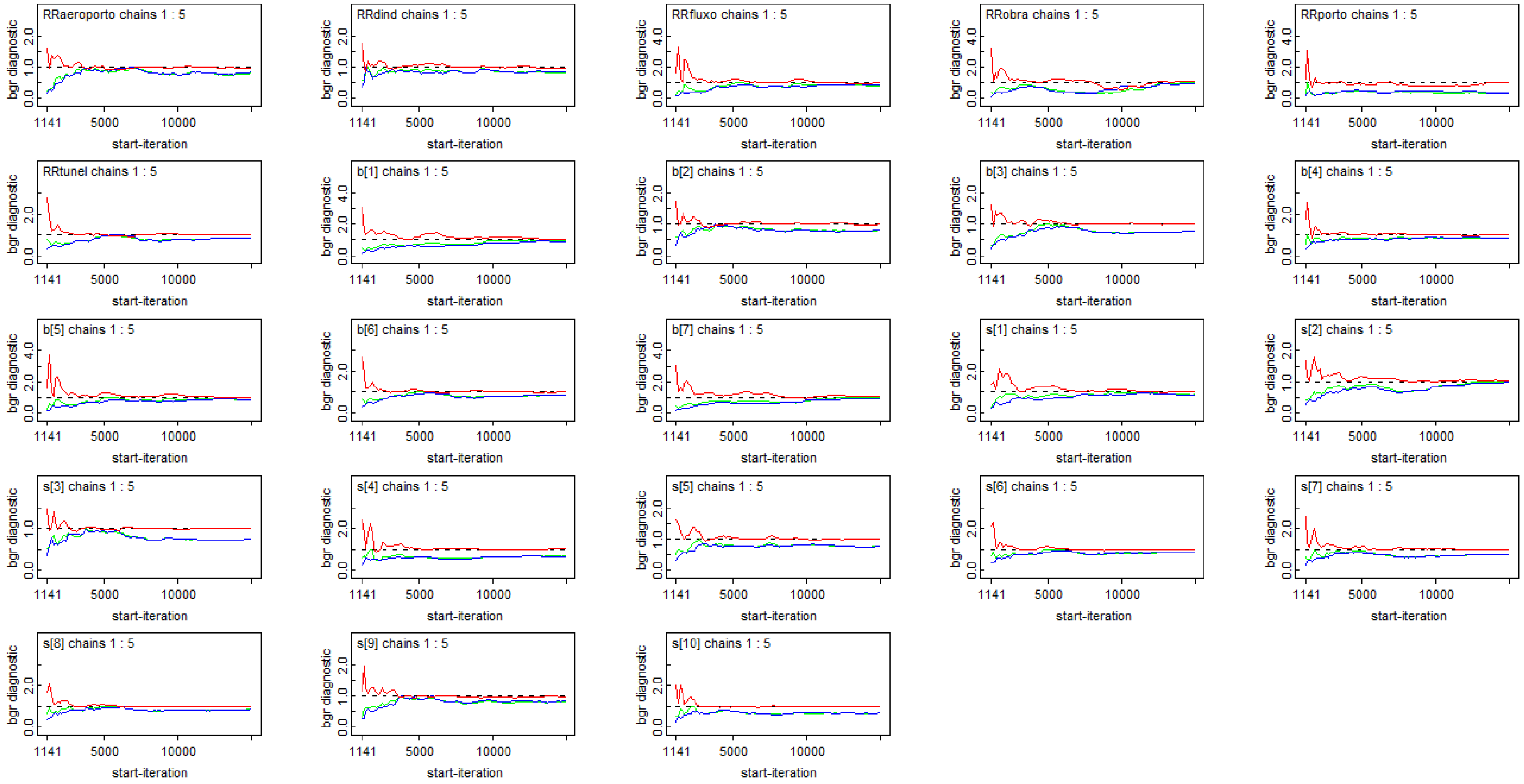

1.5. Adults (15-59 years-old)

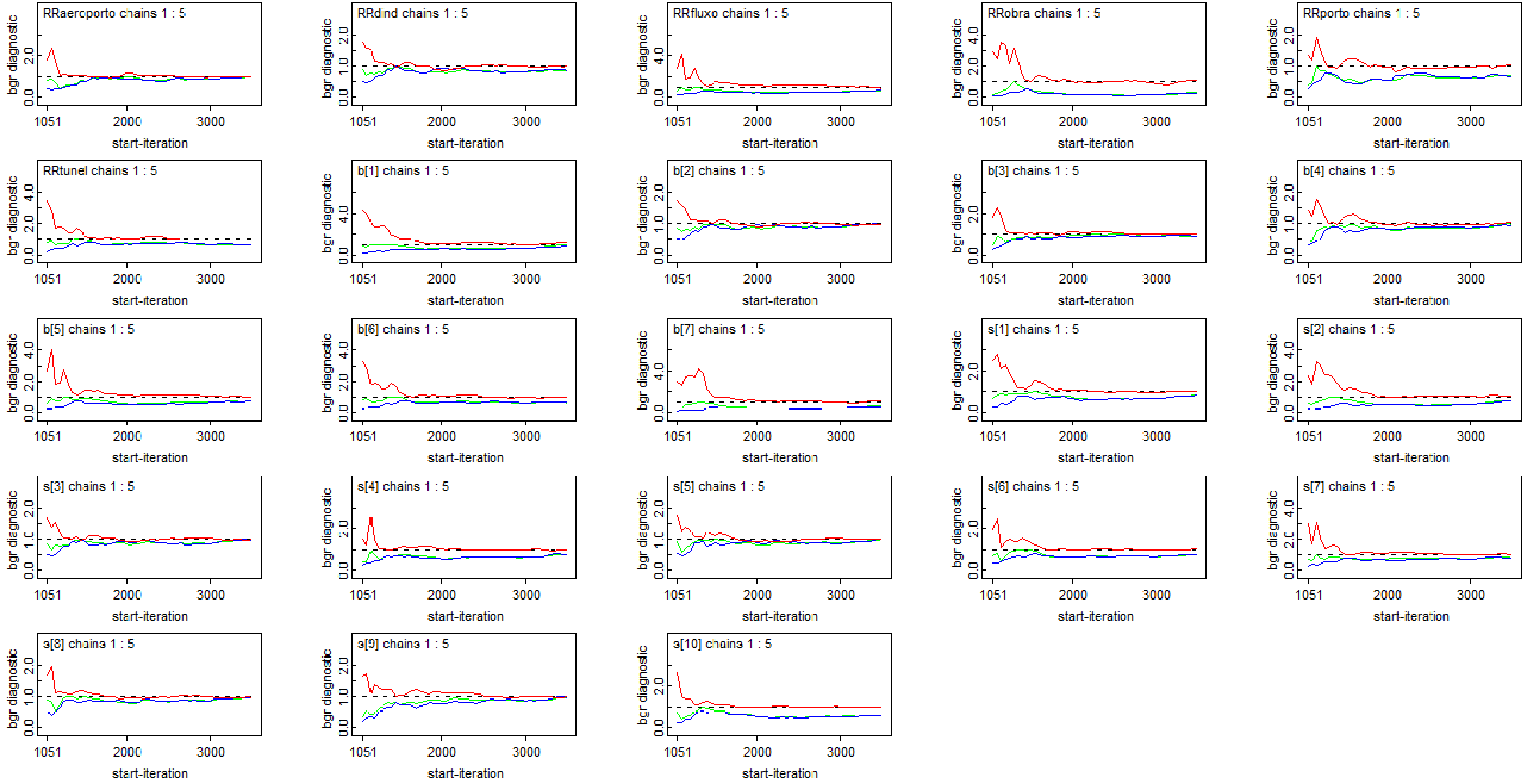

1.6. Elderly (≥60 years-old)

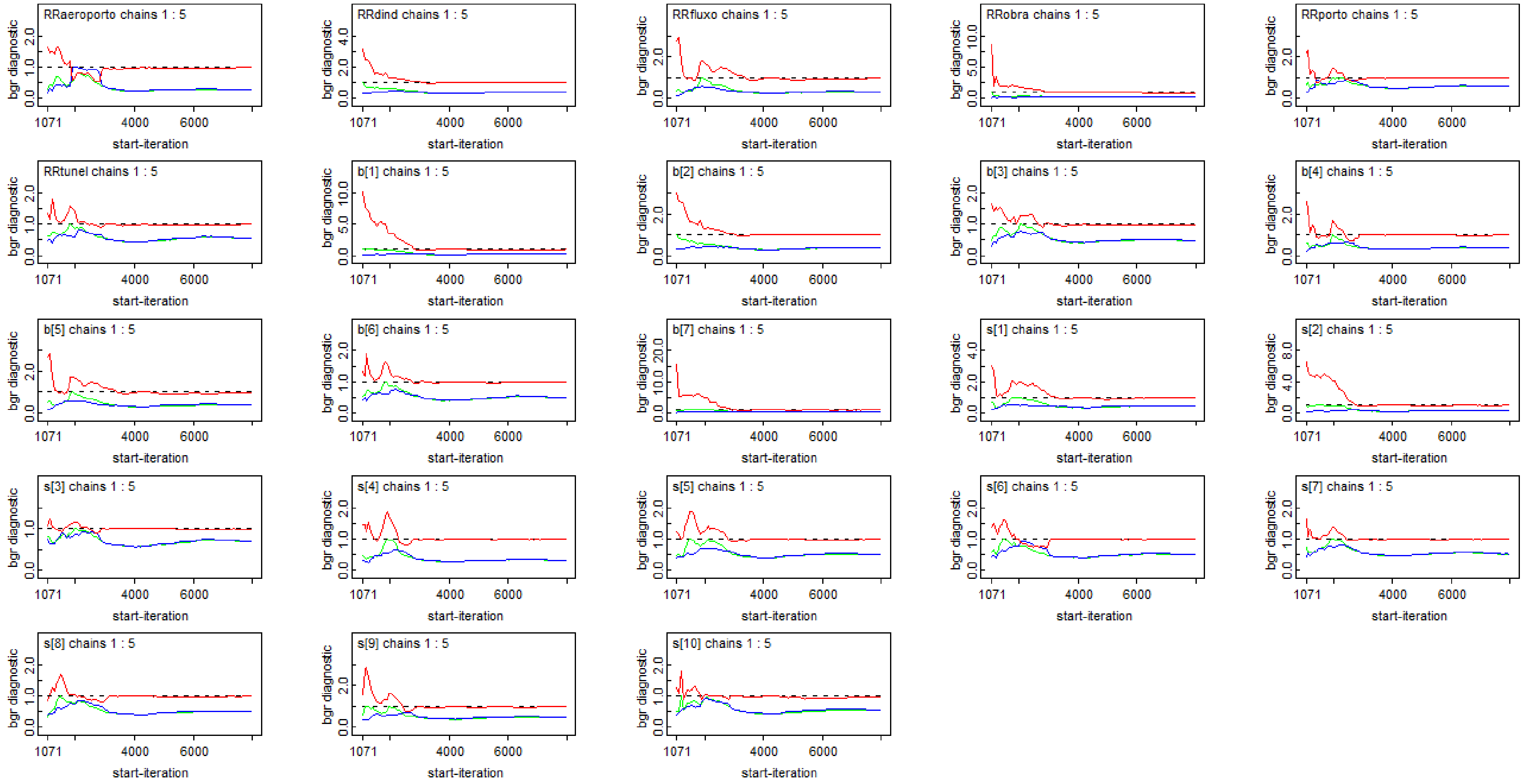

RR= estimated relative risk for each explanatory variable. b= beta regression coefficient for each explanatory variable. s= spatial component (neighborhood influence for each city planning areas). aeroporto= airport; dind= industrial district; fluxo= traffic density; obra= construction/road works; porto= seaport; tuneis= tunnel's portals.

Figure S2: Sequence charts used in descriptive analysis of hospitalization for respiratory diseases in total population and by sex, in the Rio de Janeiro city planning areas (PA), Brazil (2008-2017).

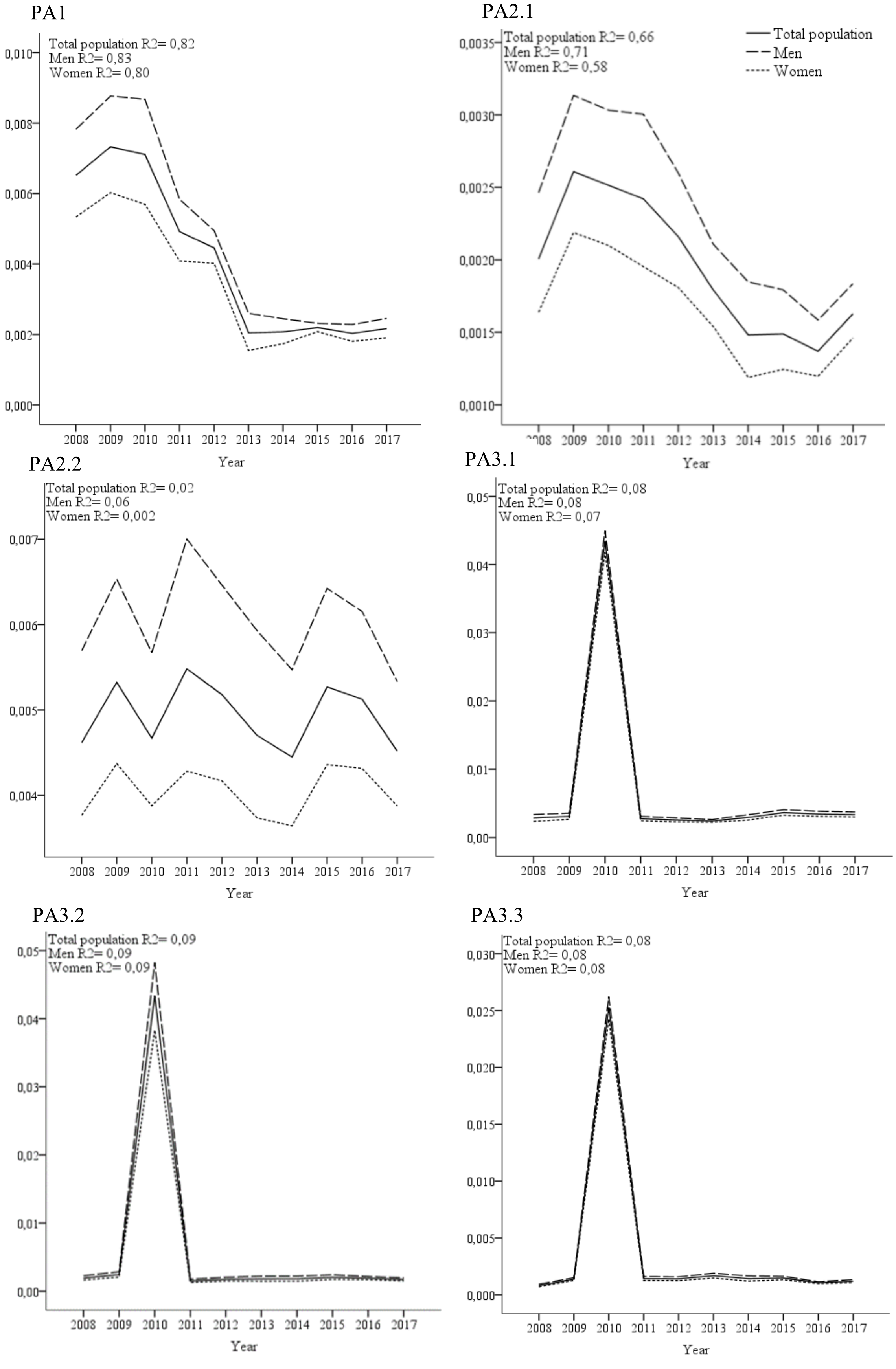

PA4

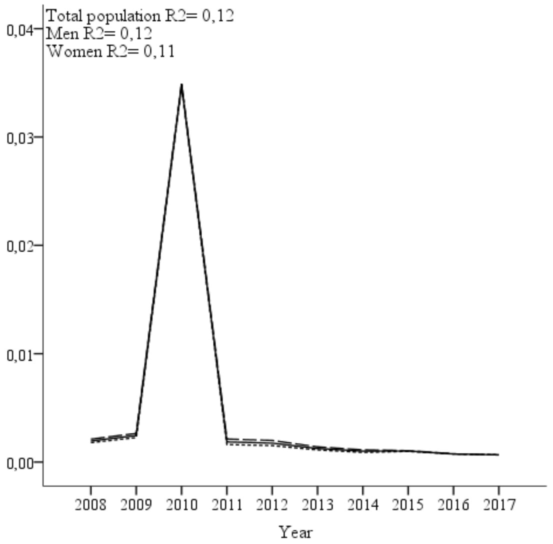

PA5.1

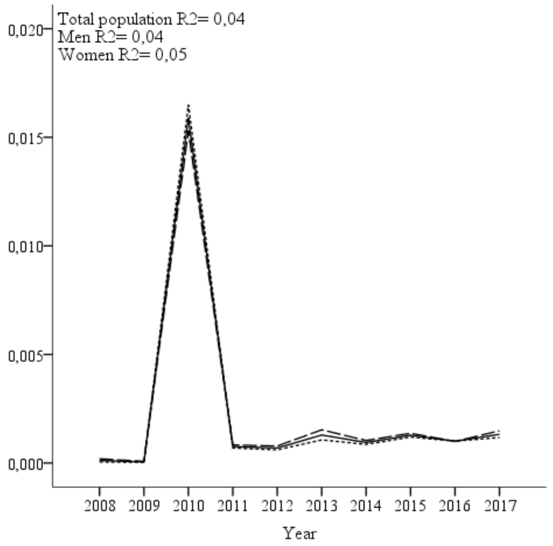

PA5.2

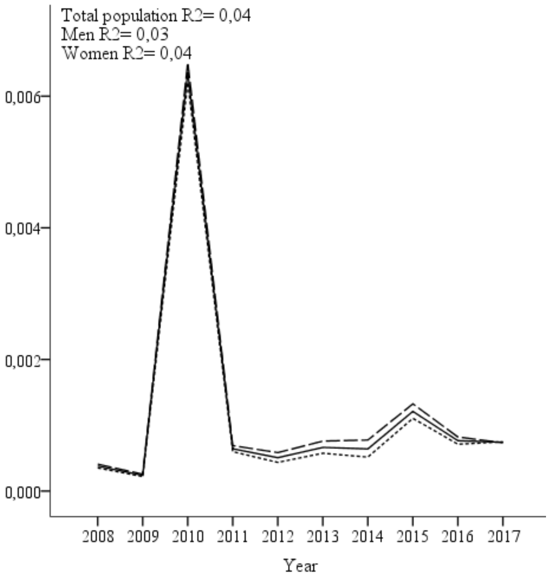

PA5.3

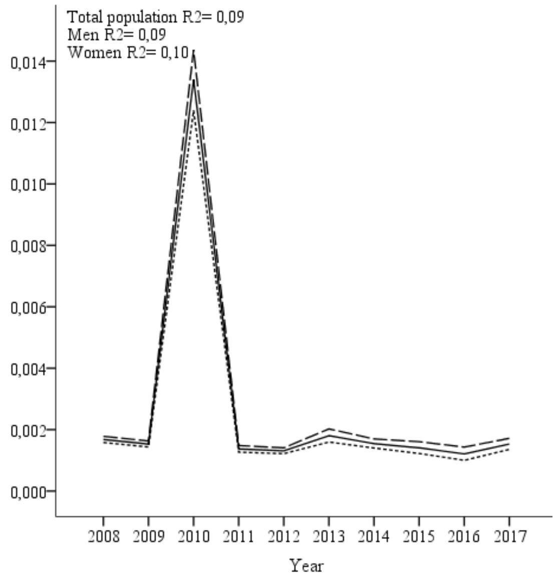

Figure S3: Sequence charts used in descriptive analysis of hospitalization for respiratory diseases by age (children: 0-4 years-old; adults: 15-59 years-old, elderly: ≥60 years-old) in the Rio de Janeiro city planning areas (PA), Brazil (2008-2017).

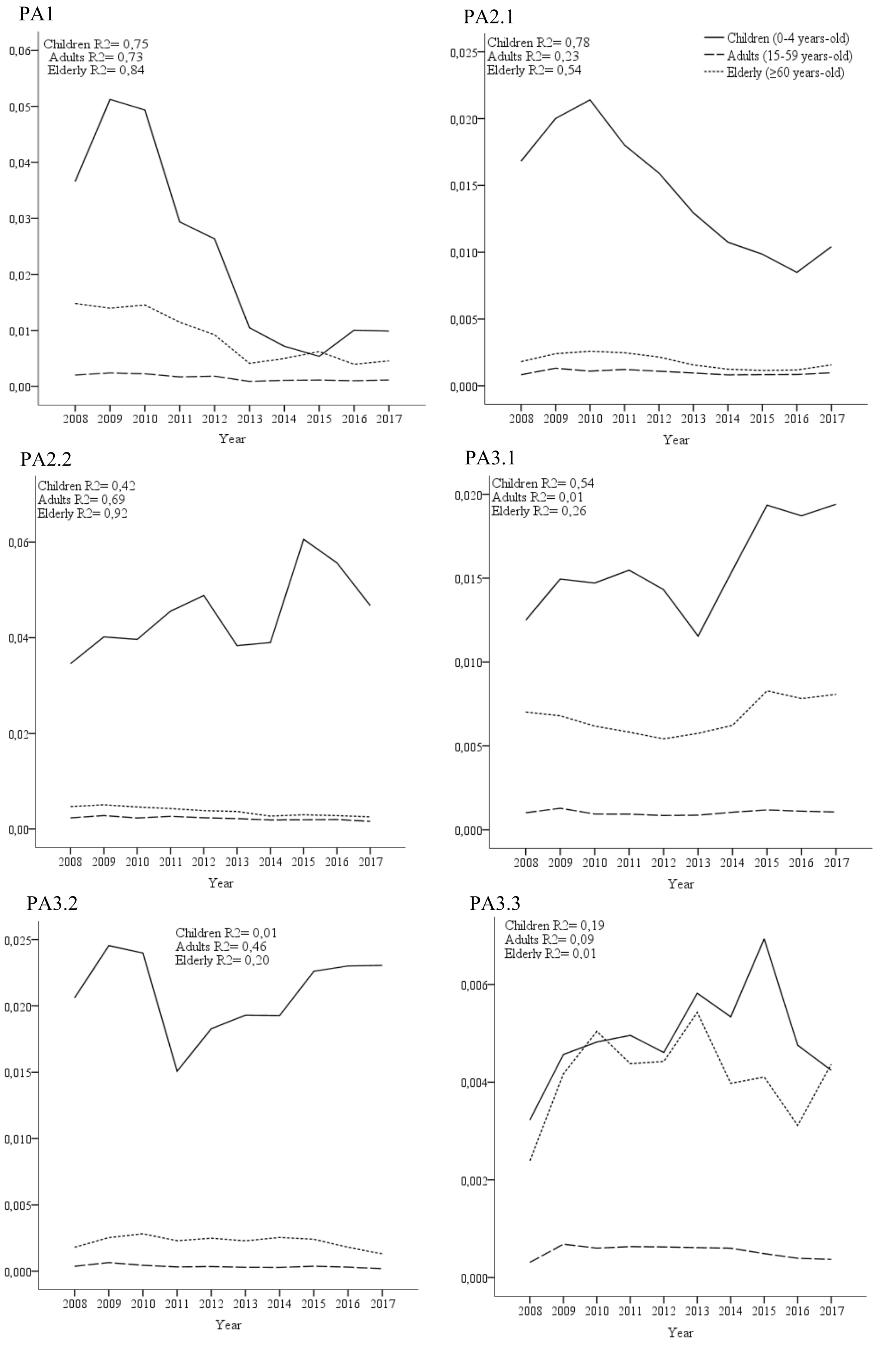

PA4

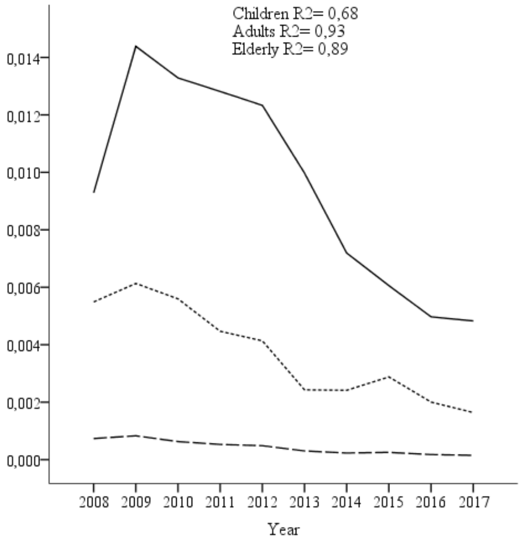

PA5.1

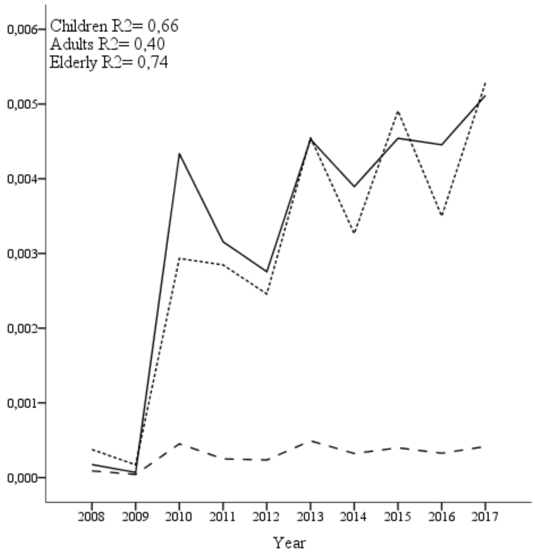

PA5.2

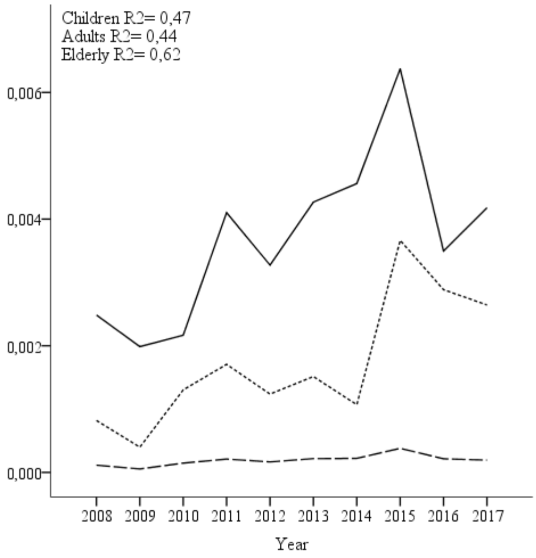

PA5.3

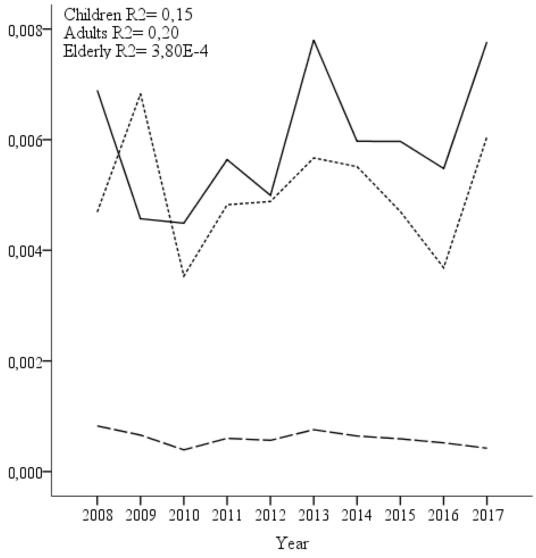

Supplement: Supplementary file 1 [file ijerph-18-04716-s001.zip › ijerph-1158236-supplementary.pdf]
